# Cancer care continuity under 2026 regional gulf conflict: experiences and a resilience framework from the Gulf States

**DOI:** 10.3389/fonc.2026.1836576

**Published:** 2026-04-23

**Authors:** Humaid O. Al-Shamsi, Saeed Rafii, Maha Al Sindi, Ahmad Alhuraiji, Nedal Bukhari, Ibrahim Abu-Gheida, Deborah Mukherji

**Affiliations:** 1 Burjeel Cancer Institute, Burjeel Medical City, Abu Dhabi, United Arab Emirates; 2 Dana-Farber Cancer Institute and Harvard Medical School, Boston, MA, United States; 3 Department of Medicine, Ras Al Khaimah Medical and Health Sciences University, Ras Al Khaimah, United Arab Emirates; 4 Emirates Oncology Society, Dubai, United Arab Emirates; 5 Department of Oncology, Mediclinic City Hospital, Dubai, United Arab Emirates; 6 Bahrain Oncology Center, Royal Medical Services, Manama, Bahrain; 7 Department of Translational Research, Dasman Diabetes Institute, Kuwait City, Kuwait; 8 Department of Hematology, Kuwait Cancer Control Center, Ministry of Health, Kuwait City, Kuwait; 9 Department of Oncology, Prince Sultan Military Medical City, Ministry of Defense Health Services, Riyadh, Saudi Arabia; 10 OncoClinic Group, Riyadh, Saudi Arabia; 11 Clemenceau Medical Centre Dubai, Dubai, United Arab Emirates

**Keywords:** cancer screening, chemotherapy, clinical trial, crises, drug delivery, radiation oncology

## Abstract

Armed conflicts and geopolitical instability pose significant risks to healthcare systems, particularly for patients with cancer who rely on uninterrupted, resource-intensive care pathways. Disruptions to diagnostic services, pharmaceutical supply chains, infrastructure, and workforce capacity have repeatedly compromised oncology care in conflict-affected settings such as Gaza, Syria, Sudan, and Ukraine. While much of the existing literature focuses on system collapse, there remains limited attention to how continuity of cancer care can be preserved during ongoing crises. Recent regional tensions in the Gulf, including missile and drone attacks targeting critical infrastructure across several Gulf Cooperation Council (GCC) countries, have raised concerns about potential healthcare disruptions. Despite these challenges, oncology services in countries such as the United Arab Emirates, Bahrain, and Kuwait have continued to operate without major interruption. Invitations to contribute were extended to oncology representatives from all six GCC countries; comprehensive accounts were received from the United Arab Emirates, Bahrain, Kuwait, and Saudi Arabia. The absence of contributions from Oman and Qatar is noted as a limitation. This Perspective examines the factors that enabled the continuity of cancer care in the Gulf during the period of instability beginning in March 2026 and considers the potential risks if the situation persists for six to twelve months. Key contributors to resilience include advanced healthcare infrastructure, robust pharmaceutical supply chains, strong governance, a highly skilled workforce, and sustained public trust in healthcare institutions. These findings highlight that resilience in oncology extends beyond system survival and reflects the system’s capacity to maintain high-quality, uninterrupted cancer care under prolonged stress. Understanding these enabling factors provides important lessons for strengthening health system preparedness and ensuring continuity of care in the face of geopolitical instability and other large-scale disruptions.

## Introduction

Cancer does not pause during war. Unlike some medical conditions that can be temporarily deferred during crises, cancer treatment follows strict biological timelines. Delays in chemotherapy, interruptions in radiotherapy schedules, or postponed oncologic surgery, even for short periods, can compromise curative outcomes and reduce survival. Armed conflicts and geopolitical instability repeatedly expose the vulnerability of healthcare systems, particularly for patients with cancer who depend on complex, multidisciplinary treatment pathways (1–3). Despite extensive documentation of healthcare disruption during war, evidence remains limited on how oncology systems can continue to operate effectively during sustained geopolitical instability.

Across several recent conflict zones, including Gaza, Syria, Ukraine, Lebanon, Yemen and Sudan, oncology services have been among the most fragile components of healthcare infrastructure (2–4). Hospitals may be damaged or overwhelmed by trauma care, supply chains for chemotherapy agents may collapse, and healthcare professionals may be displaced or unable to safely report to work (5). These disruptions frequently lead to delayed diagnoses, interrupted treatment, and poorer long-term cancer outcomes (1, 3).

The global burden of cancer continues to grow, with an estimated 20 million new cases reported worldwide in 2022, and projections indicate a significant rise in incidence over the coming decades (6). In the United Arab Emirates (UAE), early-onset cancers constitute a substantial share of cases, with approximately 25.4% diagnosed in individuals under 40 years of age and nearly half occurring before the age of 49 (7). These epidemiological patterns highlight the critical need to maintain uninterrupted cancer care, even in the context of regional or systemic instability.

Recent geopolitical instability in the Gulf region that began at the end of February 2026 and was characterized by missile and drone strikes affecting critical infrastructure in multiple countries, has heightened concerns about the resilience of civilian sectors, particularly healthcare systems and medication supply chains. Media reports during this period highlighted serious risks to pharmaceutical supply chains, including major disruptions to air cargo routes critical for the timely importation of temperature-sensitive oncology drugs (8). Nevertheless, oncology services across several Gulf nations, including the United Arab Emirates, Bahrain, Kuwait and Saudi Arabia, were maintained with minimal interruption. This context provides a valuable case study for understanding how healthcare systems can preserve the delivery of complex cancer care amidst periods of regional uncertainty and conflict.

This Perspective draws on detailed operational experiences from oncology services in the United Arab Emirates, Bahrain, Kuwait, and Saudi Arabia. Invitations to contribute were extended to oncology representatives from all six GCC countries, and comprehensive accounts were received from these four nations. The absence of contributions from Oman and Qatar is acknowledged as a limitation of the current analysis. Nevertheless, the shared regional healthcare infrastructure, regulatory environment, and socioeconomic context across the GCC support the broader applicability of the proposed Gulf Oncology Resilience Framework. This perspective provides a timely, multi-country examination of oncology service continuity and system resilience during the February 2026 Gulf regional conflict, based on operational experiences in the United Arab Emirates, Bahrain, Kuwait, and Saudi Arabia.

## The United Arab Emirates experience

Over the past two decades, the United Arab Emirates has invested heavily in developing a modern healthcare system with advanced tertiary care capacity. These investments have enabled the development of comprehensive cancer centers equipped with state-of-the-art radiotherapy technologies, imaging, laboratory and molecular diagnostics, and multidisciplinary oncology programs (7, 9).

During the recent period of regional security tensions, oncology services across the UAE continued operating without major disruption. Outpatient clinics remained open, chemotherapy infusions proceeded according to schedule, and radiotherapy departments maintained routine treatment delivery. No significant reduction in chemotherapy volumes was observed, and outpatient attendance remained stable throughout the period of instability. Oncology surgeries were performed without interruption, and multidisciplinary tumor boards continued functioning normally, ensuring that patients received timely care.

One of the key elements supporting this continuity was the resilience of the national pharmaceutical supply chain. Oncology care relies on a highly complex global network encompassing the production and distribution of chemotherapy agents, targeted therapies, immunotherapies, and supportive medications. In many conflict settings, disruptions to these supply chains quickly translate into treatment delays and drug shortages. In the UAE, however, drug importation, distribution, and regulatory oversight are coordinated through robust national frameworks that ensure sustained access to essential medications. Notably, the Emirates Drug Establishment (EDE) has convened a national task force to strengthen pharmaceutical supply chain resilience and ensure the continuous availability of essential medicines, even during periods of regional instability (10). This initiative forms part of broader efforts to strengthen institutional partnerships with stakeholders across the healthcare and logistics sectors, supporting the sustainability of pharmaceutical supplies, enhancing drug security in the country, and ensuring the continued availability of medicines and medical products to meet the needs of patients and healthcare facilities (10).

Equally important was the commitment of the healthcare workforce. Physicians, oncology nurses, pharmacists, radiation therapists, and allied health professionals continued their duties to maintain uninterrupted oncology services despite the broader regional uncertainty and active missiles and drone attacks.

In many conflict settings, patients may delay or avoid hospital visits due to safety concerns or uncertainty about service availability. In the UAE, public trust in the healthcare system meant that outpatient clinic appointments, diagnostic imaging visits, and treatment sessions continued as planned. In our experience, less than 5% of patients requested virtual consultation and follow-ups rather than attending in person due to safety concerns for themselves and their caregivers. This sustained patient engagement reflects confidence in the stability and reliability of the healthcare system.

Another important observation during this period was the ability of UAE cancer centers to accommodate patients who had previously planned to travel abroad for treatment but were unable to do so due to regional travel disruptions. Local oncology centers successfully integrated these patients into treatment pathways without delaying care, demonstrating the growing maturity of the UAE oncology ecosystem (11).

## Bahrain experience

During the recent period of regional instability, oncology services in Bahrain maintained continuity of anticancer therapy through rapid operational adaptations rather than large-scale infrastructure changes. The day-case chemotherapy and immunotherapy unit operated at near-full capacity, with infusions delivered according to established protocols. Staff schedules were adjusted to ensure consistent coverage, and contingency plans were activated to manage any fluctuations in personnel availability.

Outpatient services were quickly reconfigured into a hybrid model. Routine outpatient follow-up visits for stable patients were switched to teleconsultation platforms to reduce patient travel requirements, while in-person appointments were preserved for new referrals, symptomatic patients, or those requiring urgent care. This mixed approach integrated seamlessly into existing workflows, including multidisciplinary team discussions, and allowed continued close clinical oversight while minimizing exposure risks (12).

A temporary early decline in attendance, driven by safety concerns and movement restrictions, was reversed through proactive patient outreach via phone calls and institutional messaging, restoring activities to near-baseline levels within weeks.

Inpatient oncology services remained fully operational for admissions involving acute complications, planned intensive chemotherapy, or complex supportive care. Bed allocation for oncology patients was prioritized through close coordination with hospital leadership. Visitor policies were tightened to protect immunocompromised individuals, and alternative communication channels were established to maintain family contact. This measure was implemented in Bahrain based on local risk assessments, patient vulnerability profiles, and institutional safety evaluations for both patients and healthcare workers. Each Gulf country made context-specific operational adjustments according to its individual assessments and perceived threat levels at the time.

Pharmacy and procurement teams were central to preventing treatment interruptions. Advance prescription verification, strategic dose rounding, and vial-sharing protocols were implemented where clinically appropriate to conserve stock. For stable patients on oral targeted therapies or supportive medications, longer dispensing intervals were authorized to reduce the need for frequent hospital visits. Procurement pathways were diversified, with increased reliance on regional land transport when air cargo faced delays. Real-time inventory monitoring enabled the early identification and mitigation of potential shortages (13).

The Bahrain experience illustrates that, alongside strong baseline infrastructure, operational agility, particularly in hybrid care delivery and pharmacy stewardship, effectively safeguards oncology services during short-term regional crises. These practical adaptations complemented the resilience observed across other Gulf centers and offer actionable examples for maintaining treatment continuity under similar conditions.

## Kuwait experience

The delivery of oncology care is highly vulnerable to disruptions in infrastructure, logistics, and supply chains. On February 28, 2026, the onset of regional conflict resulted in the closure of airspace and the Strait of Hormuz, abruptly halting both air and sea cargo routes into Kuwait. This posed an immediate threat to cancer care continuity, given the country’s reliance on international supply chains for medications, consumables, and diagnostic resources.

Despite these challenges, oncology services under the Ministry of Health in Kuwait remained operational, with inpatient care, chemotherapy delivery, and management of oncologic emergencies continuing according to standard protocols. This prioritization reflects the recognition that delays in cancer treatment are associated with poorer clinical outcomes (14).

Outpatient services were rapidly restructured to reduce unnecessary hospital visits while maintaining clinical oversight. Patients on stable oral therapies were transitioned to telemedicine follow-up, leveraging systems established during the COVID-19 pandemic (15). This approach minimized patient exposure risks while preserving healthcare resources.

Pharmaceutical supply disruptions represented one of the most immediate challenges. To preserve national stock, medication dispensing was limited to defined intervals, while alternative procurement strategies were implemented, including rerouting shipments through neighboring countries. Collaboration with regional logistics networks and Kuwait’s national carriers played a key role in maintaining access to essential oncology medications from global inventory.

Resource constraints also affected the availability of consumables and diagnostic services. Shortages of key items, such as vascular access devices prompted stricter utilization protocols, ensuring that limited items were reserved for patients with the highest clinical need. Similarly, advanced diagnostic testing, including molecular and cytogenetic analyses, was prioritized for essential clinical decision-making, while routine follow-up testing was adapted based on clinical necessity.

The Kuwait experience underscores the importance of preparedness, adaptability, and regional cooperation in sustaining oncology care during crises. Strategies developed during the COVID-19 pandemic, particularly telemedicine integration, resource prioritization, and flexible care delivery models, proved highly transferable.

Overall, despite significant logistical constraints, oncology services in Kuwait remained resilient. This experience highlights the critical need for diversified supply chains, adaptive clinical pathways, and coordinated national and regional strategies to ensure the continuity of cancer care during periods of geopolitical instability.

## Saudi Arabia experience

Saudi Arabia’s experience during the recent period of regional instability demonstrates that oncology services continued seamlessly, with no disruption to healthcare delivery across the Kingdom. The country’s large territorial scale and well-structured healthcare system supported the uninterrupted functioning of healthcare services nationwide.

The oncology system in Saudi Arabia is characterized by a well-established and geographically distributed network of cancer centers across cities throughout the country. This decentralized structure ensures continuous delivery of care, with all services proceeding as scheduled across the national network. Major tertiary institutions, including specialized oncology centers under the Ministry of Health and other healthcare sectors, maintained full operational capacity, consistently providing chemotherapy, radiotherapy, and surgical oncology services.

Saudi Arabia’s robust logistical infrastructure further supports the continuity of oncology care. Multiple international airports across different regions, along with access to both the Arabian Gulf and the Red Sea, provide stable and diversified supply routes. This logistical strength ensures reliable pharmaceutical supply chains, with uninterrupted availability of essential oncology medications, including chemotherapy and targeted therapies.

In addition, the national healthcare framework, supported by centralized coordination and sustained investment in infrastructure, facilitates consistent system performance. Digital health platforms and integrated healthcare systems enable efficient coordination between institutions, supporting smooth patient management and optimal resource allocation.

Overall, oncology services in Saudi Arabia have remained fully operational and stable across all cities, reflecting the strength of a well-planned, decentralized, and resilient healthcare system capable of sustaining uninterrupted cancer care under all circumstances.

## Psychological well-being of cancer patients and caregivers during regional instability

Over 30% of cancer patients already experience substantial psychological distress even under baseline conditions (16). During the recent Gulf regional tensions, our daily interactions with patients and their caregivers revealed that, in some individuals, this burden was further amplified by fears of treatment interruption, disease progression, and threats to personal safety.

In the UAE, Bahrain, and Kuwait, multidisciplinary oncology teams proactively maintained and expanded psychosocial support services. Existing psycho-oncology programs continued without interruption, delivering routine mental health screening at every outpatient visit and infusion, with immediate referrals to counsellors or support groups, particularly in larger hospitals. Tele-oncology platforms facilitated virtual counselling sessions, allowing patients to receive support from home while reducing travel-related stress. Caregivers, many managing their own safety concerns, were offered dedicated family sessions and peer-support networks.

Some hospitals used text messages to communicate the continuation of oncology services. These consistent, transparent updates played a pivotal role in mitigating fear, restoring confidence, and sustaining treatment adherence.

This experience demonstrates that psychological resilience is not incidental but an essential pillar of oncology system resilience. By embedding mental health support within the broader framework, Gulf oncology centers ensured that patients did not face the “dual fronts” of physical disease and unaddressed emotional trauma simultaneously (17).

## Scenario analysis: if regional conflict persists for 6–12 months

Unlike acute conflict disruptions, prolonged instability creates cumulative and compounding risks to oncology systems. While the immediate continuity of oncology services during short periods of geopolitical instability demonstrates important health system resilience, a prolonged conflict lasting six months or longer would introduce a different set of challenges for cancer care systems, **Table 1**. Unlike short-term disruptions, extended periods of instability may progressively affect healthcare infrastructure, workforce capacity, diagnostic and pharmaceutical supply chains, and patient access to care. Understanding these potential risks is essential for proactive preparedness and strategic planning.

**Table 1:** Short-term vs prolonged conflict risks for oncology systems.

Dimension	Short-term conflict (days–weeks)	Prolonged conflict (6–12 months)
Drug Supply	Stockpiles maintain treatment continuity	Risk of anticancer therapies shortages and treatment discontinuation, delay or modification
Diagnostic supplies	Stockpiles maintain services, minimal disruption	Risk of shortages, shelf life and expiry dates of consumables, regular servicing and calibration of equipment
Radiotherapy	Minimal disruption	Equipment maintenance challenges and treatment disruption
Workforce	Staff remain operational	Burnout, mobility concerns, potential workforce attrition
Patient Behavior	Continued treatment adherence	Delayed diagnosis, treatment and reduced screening participation
Healthcare Financing	Minimal impact	Potential pressure on insurance and healthcare budgets, fund allocation
Clinical Trials	Impact on transportation of samples abroad due to air travel disruption	protocol deviations, prematurely termination of active clinical trials, suspending the opening of new trials
Patient Movement	Regional travel disruption	Increased local demand and cross-border patient flow

Importantly, the 2026 Gulf experience occurred amid regional tensions involving targeted infrastructure threats and temporary airspace and shipping disruptions rather than the widespread, sustained high-intensity combat and total supply-chain severance documented in settings such as Sudan or historical conflicts in Iraq. In those higher-intensity contexts, rapid and extensive destruction of healthcare facilities, complete supply-chain collapse, and mass displacement frequently reduce the oncology resilience window from months to weeks, shifting priorities from maintenance of full-spectrum curative care to emergency triage and palliative measures. The Gulf cases therefore illustrate system resilience under moderate but protracted disruption, offering complementary lessons for different conflict intensities and durations.

This table summarizes the differential impact of short-term versus prolonged conflict on key domains of oncology care. While short-term disruptions are often mitigated by existing system capacity, prolonged instability leads to cumulative challenges affecting drug supply, workforce stability, service delivery, and patient outcomes.

One of the most immediate vulnerabilities during prolonged conflict is the stability of oncology drug supply chains. Cancer treatment relies on complex international manufacturing and distribution networks involving chemotherapy agents, targeted therapies, immunotherapies, and supportive medications. Although short-term stockpiles may buffer temporary disruptions, extended geopolitical instability can affect global shipping routes, air cargo availability, and pharmaceutical production (13). These disruptions may lead to shortages of critical oncology medications, forcing clinicians to modify treatment protocols, delay cycles of chemotherapy, or substitute less optimal regimens. Experiences from conflict-affected regions such as Ukraine and Syria have demonstrated that prolonged disruptions in medication availability can significantly compromise treatment outcomes for cancer patients (3, 18).

Another critical vulnerability during extended conflict is the sustainability of radiotherapy infrastructure. Radiation oncology services depend on highly specialized equipment that requires a continuous electricity supply, regular technical maintenance, and access to replacement parts from international manufacturers. Prolonged disruptions to logistics or engineering support could affect the ability of radiotherapy centers to maintain uninterrupted treatment schedules. Even minor interruptions can result in treatment delays that affect tumor control, particularly for curative treatment protocols in cancers such as head and neck, cervical, and lung cancer. To mitigate these risks, several practical strategies can be implemented. Ensuring redundant and resilient power supply systems, including backup generators and uninterruptible power supply (UPS) units is essential to maintain uninterrupted machine operation. Preventive maintenance programs should be intensified, with pre-emptive servicing and the stockpiling of critical spare parts to reduce dependence on international supply chains (19). Establishing local or regional technical support capabilities, including training biomedical engineers, can further enhance rapid response to equipment failures. In parallel, developing contingency treatment pathways, such as redistributing patients across a network of radiotherapy centers or utilizing alternative fractionation schedules when clinically appropriate, can help sustain treatment delivery (20). Strengthening digital infrastructure for remote diagnostics and vendor support also allows real-time troubleshooting, minimizing downtime.

The sustainability of the oncology workforce becomes increasingly critical during prolonged crises. Cancer care depends on highly specialized multidisciplinary teams, including medical and radiation oncologists, oncology nurses, pharmacists, medical physicists, and technical staff. Across many Gulf healthcare systems, a substantial proportion of this workforce consists of expatriate, non-GCC professionals (21). During periods of geopolitical instability, this reliance introduces an additional layer of vulnerability, as concerns related to personal and family safety, travel restrictions, or uncertainty regarding long-term residency may influence decisions to temporarily relocate or leave the region.

Beyond workforce mobility, extended crises can also contribute to cumulative burnout and psychological stress among healthcare professionals, who must balance demanding clinical responsibilities with personal concerns about regional security. These factors may collectively impact workforce stability and continuity of care if not proactively addressed (22).

Oncology staff across the Gulf have demonstrated a high level of dedication and commitment to the healthcare systems they serve. Nonetheless, to mitigate the risks outlined above, several strategies are essential. Strengthening workforce retention policies, including clear communication, reassurance regarding safety measures, and institutional support for staff and their families, can help maintain confidence and reduce attrition. Providing access to mental health and psychosocial support services is equally important to address burnout and stress. Developing local workforce capacity through sustained investment in national training programs and oncology fellowships can reduce long-term reliance on expatriate staff. In parallel, implementing flexible staffing models and cross-training within multidisciplinary teams can enhance operational resilience. Finally, establishing regional collaboration frameworks and contingency staffing plans, including temporary credentialing pathways and inter-institutional support agreements, can help ensure continuity of oncology services even in the event of workforce fluctuations.

Beyond healthcare infrastructure and workforce considerations, prolonged conflict may also influence patterns of cancer diagnosis and disease presentation (3). In many conflict settings, preventive services and cancer screening programs are often among the first healthcare activities to decline. Public anxiety, transportation disruptions, and competing healthcare priorities may discourage individuals from seeking medical attention for early symptoms. Over time, this can lead to a decline in early-stage diagnoses and an increase in patients presenting with more advanced disease (14). This phenomenon, often described as “stage migration,” has been documented in multiple conflict-affected regions and can significantly affect long-term cancer survival outcomes (3).

Economic factors may also emerge as an additional pressure point for oncology care systems during prolonged instability. Cancer treatment is resource-intensive, often involving expensive systemic therapies, advanced imaging, and complex multidisciplinary care (23). If prolonged geopolitical instability affects economic activity or insurance coverage, healthcare systems may face increasing financial pressure to sustain access to high-cost treatments such as immunotherapy, targeted therapies, and advanced cellular therapies. Maintaining equitable access to modern cancer treatments therefore requires strong healthcare financing frameworks capable of absorbing such pressures.

Another potential consequence of prolonged regional instability is changes in patterns of cross-border patient movement. The Gulf region has historically served as a hub for medical travel, with many patients seeking specialized oncology care across national borders (24). If travel to distant international centers becomes restricted during prolonged conflict, regional cancer centers in the Gulf may experience increased demand from patients who would otherwise have sought treatment abroad. While this trend reflects growing confidence in regional oncology capabilities, it may also place additional pressure on existing healthcare resources if patient volumes increase substantially. The UAE, for example, reported the ability to absorb and manage patient influx from abroad to the UAE during the COVID-19 pandemic (11).

Prolonged crises can significantly disrupt clinical trial activity, affecting patient access to innovative therapies and the generation of high-quality evidence. Trial enrollment may decline due to safety concerns, patient mobility restrictions, and reduced hospital capacity. Ongoing studies may face protocol deviations, delays in follow-up assessments, or challenges in drug supply and monitoring. In some cases, trials may be temporarily suspended or prematurely terminated due to logistical or regulatory constraints (25). These disruptions can compromise data integrity and delay the development of new treatments. Ensuring trial continuity requires adaptive protocols, remote monitoring strategies, regulatory flexibility, and robust contingency planning to safeguard both patient safety and research integrity.

Taken together, these potential challenges highlight that the resilience of oncology systems cannot be measured solely by their ability to withstand short-term disruptions. Instead, true resilience requires preparedness for prolonged periods of instability.

The experience of healthcare systems in the Gulf region during recent regional tensions provides an opportunity to consider how oncology services can prepare for extended crisis scenarios. By anticipating potential vulnerabilities and implementing proactive mitigation strategies, healthcare systems can ensure that cancer care continues even during prolonged geopolitical uncertainty. In doing so, oncology services become not only a component of healthcare delivery but also a critical pillar of national health security.

## Cancer care as critical national security infrastructure

Recognizing cancer care infrastructure as a strategic national asset highlights the importance of coordinated planning between healthcare authorities, emergency preparedness agencies, and national security institutions. Integrating healthcare resilience into national crisis management strategies can help ensure that essential medical services remain operational during periods of geopolitical instability (26).

The experience of Gulf healthcare systems during recent regional tensions demonstrates how investments in healthcare infrastructure, regulatory oversight, and emergency preparedness frameworks can contribute to protecting complex medical services such as oncology care (27). As global geopolitical risks continue to evolve, incorporating healthcare system protection into national security strategies may become increasingly important. Failure to maintain cancer care during conflict may result in a secondary, delayed mortality burden that exceeds the immediate impact of the crisis itself (28).

## A gulf oncology resilience framework

**Figure 1:**
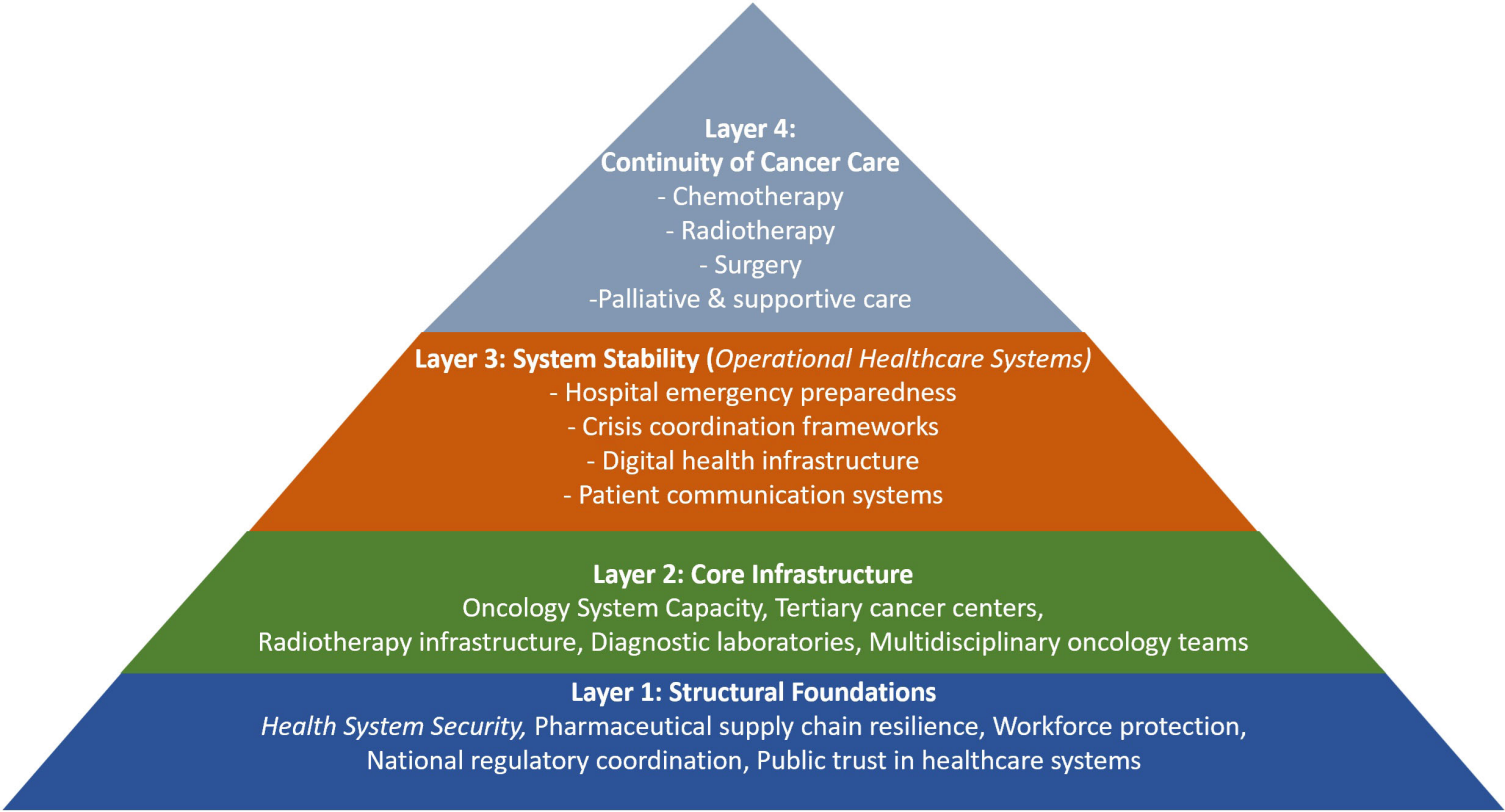
Framework for oncology system resilience during conflict and prolonged instability. This conceptual model illustrates the structural components required to maintain oncology services during periods of geopolitical instability. The resilience of cancer care systems depends on the interaction between healthcare infrastructure, pharmaceutical supply security, workforce stability, governance structures, and sustained patient trust. Together, these elements enable the continuity of complex oncology treatments even during periods of crisis.

The experience of healthcare systems in the Gulf region suggests that oncology system resilience during periods of instability is supported by several interconnected pillars. These elements collectively form a framework that may guide preparedness planning in other regions facing potential crises. **Figure 1** illustrates the structural components required to maintain oncology services during periods of geopolitical instability. The model is presented as a hierarchical pyramid, demonstrating increasing resilience from foundational elements to clinical outcomes.

In all participating Gulf countries, established digital health platforms were instrumental in sustaining care continuity. In the UAE, these were underpinned by national-scale electronic health record (EHR) systems that enabled seamless cross-facility data sharing, treatment scheduling, and multidisciplinary team coordination. Bahrain and Kuwait leveraged telemedicine platforms originally scaled during the COVID-19 pandemic, incorporating secure video consultations, electronic prescribing, remote laboratory result review, and basic symptom-monitoring modules (29, 30). While advanced automated triage or real-time adverse-event tracking modules were not universally implemented at the time of the crisis, the existing infrastructure allowed rapid transition to hybrid care models, reduced unnecessary hospital visits, and preserved clinical oversight for stable patients. These experiences underscore the value of fully integrated national EHR systems with oncology-specific functionalities as a core component of future health-system resilience planning.

## Global lessons from conflict-affected oncology systems

The experience of oncology care delivery during recent regional instability, together with evidence from conflict-affected settings globally, highlights the need to reposition cancer care as a core component of health system resilience and national security planning. Traditionally, emergency preparedness frameworks have prioritized acute and communicable conditions, often overlooking chronic, resource-intensive diseases such as cancer. However, cancer remains substantially under-integrated into humanitarian response frameworks despite affecting large populations in fragile and conflict-affected settings, underscoring a critical gap in global health policy (26).

Importantly, the 2026 Gulf experience occurred amid regional tensions involving targeted infrastructure threats and temporary airspace/shipping disruptions rather than the widespread, sustained high-intensity combat and total supply-chain severance documented in settings such as Sudan or historical conflicts in Iraq. In those higher-intensity contexts, rapid and extensive destruction of healthcare facilities, complete supply-chain collapse, and mass displacement frequently reduce the oncology resilience window from months to weeks, shifting priorities from maintenance of full-spectrum curative care to emergency triage and palliative measures. The Gulf cases therefore illustrate system resilience under moderate but protracted disruption, offering complementary lessons for different conflict intensities and durations.

Health policy frameworks must therefore adopt a more integrated approach that embeds the full cancer care continuum, including prevention, early detection, treatment, and palliative care, within national emergency preparedness and response strategies. Recent global policy recommendations emphasize integrating cancer into preparedness plans, strengthening surveillance systems, and improving coordination across health systems to enhance resilience and continuity of care during crises (26). In parallel, strengthening pharmaceutical supply chains and protecting healthcare infrastructure are essential, as conflict has been shown to disrupt cancer medicine availability and displace vulnerable patient populations (8, 31).

At a systems level, establishing regional and international collaboration mechanisms is essential to support continuity of care, including cross-border referral pathways, shared procurement strategies, and coordinated responses to drug shortages. Global initiatives and policy frameworks increasingly call for international cooperation to maintain cancer services in crisis settings and to develop context-specific solutions for both acute and protracted conflicts.

Finally, incorporating oncology care into the broader global health security agenda is critical. Failure to maintain cancer services during prolonged crises risks generating a secondary wave of preventable morbidity and mortality, particularly as fragile health systems face compounded pressures from displacement, infrastructure damage, and resource constraints. Policymakers should therefore recognize that protecting cancer care is not only a clinical priority but also a strategic investment in population health resilience and long-term system stability.

## Conclusion

As global health systems confront an era increasingly shaped by geopolitical conflict, pandemics, and other large-scale disruptions, safeguarding cancer care must be recognized as a strategic priority within national and global health security frameworks. Ensuring continuity of oncology care during crises is not simply a logistical challenge but a fundamental responsibility of resilient health systems.

The experience of healthcare systems in the Gulf region demonstrates that complex oncology services can be sustained even during periods of heightened regional tension. This resilience reflects the combined strength of robust healthcare infrastructure, coordinated governance, secure pharmaceutical supply chains, a committed multidisciplinary workforce, and sustained public trust. Together, these elements form the foundation of a system capable of maintaining high-quality cancer care under external stress.

However, resilience must extend beyond the immediate response to short-term disruption. The greater challenge lies in sustaining oncology services during prolonged instability, where cumulative pressures on supply chains, workforce stability, infrastructure, and financing can erode system capacity over time. Addressing these risks requires proactive planning, strategic investment in redundancy, protection of healthcare personnel, and strengthened regional and international collaboration.

Recent international initiatives have further outlined key priorities, including advocating for full adherence to the Geneva Convention in protecting medical personnel and patients with cancer, integrating cancer and other noncommunicable diseases into UN humanitarian agendas, establishing a WHO-led working group on cancer care in conflict zones, developing inclusive care models tailored to patient needs, and creating digital platforms to enhance coordination and financing of oncology services (26). Failure to do so risks creating a secondary, preventable burden of cancer-related morbidity and mortality that may exceed the immediate impact of the crisis itself. Protecting the continuity of oncology care is therefore not only a clinical necessity, but an ethical imperative, ensuring that patients with cancer are not among the hidden casualties of conflict.

## Data Availability

The original contributions presented in the study are included in the article/supplementary material. Further inquiries can be directed to the corresponding author.
